# The Interplay between Toxic and Essential Metals for Their Uptake and Translocation Is Likely Governed by DNA Methylation and Histone Deacetylation in Maize

**DOI:** 10.3390/ijms21186959

**Published:** 2020-09-22

**Authors:** Sarfraz Shafiq, Asim Ali, Yasar Sajjad, Qudsia Zeb, Muhammad Shahzad, Abdul Rehman Khan, Rashid Nazir, Emilie Widemann

**Affiliations:** 1 Department of Anatomy and Cell Biology, University of Western Ontario, 1151 Richmond St, London, ON N6A5B8, Canada; 2 Department of Environmental Sciences, COMSATS University Islamabad, Abbottabad Campus, University Road, tobe Camp, Abbottabad 22060, Pakistan; zeb_qudsia@yahoo.com (Q.Z.); mshahzad@cuiatd.edu.pk (M.S.); rashidnazir@cuiatd.edu.pk (R.N.); 3 Department of Biotechnology, COMSATS University Islamabad, Abbottabad Campus, University Road, Tobe Camp, Abbottabad 22060, Pakistan; asim21pk@hotmail.com (A.A.); yasarsajjad@cuiatd.edu.pk (Y.S.); arehman@cuiatd.edu.pk (A.R.K.); 4 Department of Biology, University of Western Ontario, 1151 Richmond St, London, ON N6A5B8, Canada; ewidema4@uwo.ca

**Keywords:** phytotoxcity, heavy metals interplay, Zn transporters (*ZIP*), DNA methylation, histone deacetylases, maize

## Abstract

The persistent nature of lead (Pb) and cadmium (Cd) in the environment severely affects plant growth and yield. Conversely, plants acquire zinc (Zn) from the soil for their vital physiological and biochemical functions. However, the interplay and coordination between essential and toxic metals for their uptake and translocation and the putative underlying epigenetic mechanisms have not yet been investigated in maize. Here, we report that the presence of Zn facilitates the accumulation and transport of Pb and Cd in the aerial parts of the maize plants. Moreover, the Zn, Pb, and Cd interplay specifically interferes with the uptake and translocation of other divalent metals, such as calcium and magnesium. Zn, Pb, and Cd, individually and in combinations, differentially regulate the expression of DNA methyltransferases, thus alter the DNA methylation levels at the promoter of *Zinc-regulated transporters, Iron-regulated transporter-like Protein* (*ZIP*) genes to regulate their expression. Furthermore, the expression of histone deacetylases (*HDACs*) varies greatly in response to individual and combined metals, and *HDACs* expression showed a negative correlation with *ZIP* transporters. Our study highlights the implication of DNA methylation and histone acetylation in regulating the metal stress tolerance dynamics through Zn transporters and warns against the excessive use of Zn fertilizers in metal contaminated soils.

## 1. Introduction

Plants are exposed to diverse fluctuating environmental challenges during their development that can affect their fitness, survival and yield [1]. Among these challenges, crop plant exposure to various heavy metals in the soil is one of the leading factors that impairs morphological, physiological, biochemical, and molecular processes in plants, thus contributes to the reduction of yield [1,2]. The impacts of cadmium (Cd), lead (Pb), mercury (Hg), and chromium (Cr) heavy metals on plant physiology, biochemical processes, and yield have been extensively studied in plants [3,4,5]. Apart from these heavy metals, plants acquire essential metals such as iron (Fe), zinc (Zn), manganese (Mn), copper (Cu) and nickel (Ni) from the soil for their vital physiological and biochemical functions during their development [6,7]. However, the excess of these essential metals is toxic to the plants. Zn and Fe are essential for plant metabolism and are required in a precise amount for proper development. Zn and Fe deficiency causes the reduction of plant growth, yield, and grain quality in cereals [8,9,10]. However, the excess of Zn and Fe in plants may cause toxicity to the biological system [8,11]. Therefore, plants have evolved a sophisticated system to balance the uptake, storage, and utilization of these metals.

Essential metals are distributed to different cells and organelles of plants depending on their needed concentration, through a variety of metal transporters [12]. Among them, the Zinc-regulated transporters, Iron-regulated transporter-like Protein (ZIP) family is reported in crops and required for the uptake of Zn [13,14,15]. However, the *ZIP* family members may also uptake Fe, Mn, and Cd [13,16,17]. Maize has nine *ZIP* transporters [17,18,19]. Maize *ZIP* transporters are localized in plasma membrane and endoplasmic reticulum, and *ZIPs* showed differential expression under Zn and Fe treatment [17]. This suggests that maize *ZIPs* could be functional transporters of Zn and Fe. Therefore, the exploitation of maize *ZIPs* would provide an excellent tool to maintain the desired amount of essential Zn and Fe in grains, which would be helpful to address the sustainable solution of malnutrition as well as to ensure the desired crop yield.

In response to fluctuating environmental and developmental cues, the gene expression is regulated by chromatin architecture, thus controls various cellular and physiological processes [20]. In this regard, DNA methylation is associated with the gene silencing involved in different biological processes, including flowering time, imprinting, flower and leaf morphogenesis, and fertility [21,22,23]. Maize putative DNA methyltransferases have shown differential expression in response to polyethylene glycol (PEG) and sodium chloride (NaCl) treatment [24], suggesting the involvement of DNA methylation in response to environmental stress. Pb, Cd, Zn, and Ni have been shown to induce specific DNA methylation changes in wheat, white clover, industrial hemp plants, oil seed rape, and radish [25,26,27,28]. However, the effect of heavy metal stress on DNA methylation and the underlying epigenetic mechanism in crop plants, especially in maize, is poorly understood.

DNA methylation cross-talks with histone acetylation for gene expression regulation [29]. Histone acetylation is another chromatin modification, which is associated with transcriptional activation [20]. Histone acetylation homeostasis is achieved through the action of histone acetyltransferases (HATs) and histone deacetylases (*HDACs*). Histone deacetylation has been reported to play an important role in plant growth and development, flowering, seed development, and to deal with biotic stress and abiotic stress including salt, cold, and drought stresses [30,31,32]. Pb, Cd, and Zn alter the gene expression of HATs and *HDACs* in cotton [33,34], indicating their important role in other crops. Maize *HDACs* play a role in plant development [35,36,37,38]. However, the effect of heavy metals on histone acetylation in maize as well as crop plants, especially the function of maize histone deacetylases in the tolerance of metal stress, has not been studied yet. In addition, the eventual interplay and coordination among Zn, Pb, and Cd for their uptake and/or translocation and the potential involvement and epigenetic regulation of *ZIP* transporters in this particular interplay has not been investigated yet. We hypothesized that divalent metals (Pb, Cd, and Zn) interfere with each other’s uptake and translocation due to the un-specific nature of divalent Zn transporters. Further, we also hypothesized that heavy metals alter the epigenetic landscape of maize plants, which in return regulates the expression of Zn transporters.

By applying several metals to the maize plants, we found that Zn, Pb, and Cd combinations not only interfere with each other’s accumulation and mobility to aerial parts, but also interfere with the uptake and translocation of other divalent metals (calcium and magnesium). Moreover, our results indicate that the interplay among Zn, Pb, and Cd is regulated by *ZIP* transporters, which are under the control of DNA methylation and histone acetylation.

## 2. Results

### 2.1. Zn Favors the Accumulation and Mobility of Pb/Cd to the Aerial Parts of Maize Plants

As expected, we found that the exposure of plants to Pb, Cd, or Zn leads to an accumulation of the respective metals into the roots, shoots, and leaves (Table 1), showing that each metal is imported into the root and transported toward the aerial parts.

**Figure 1:**
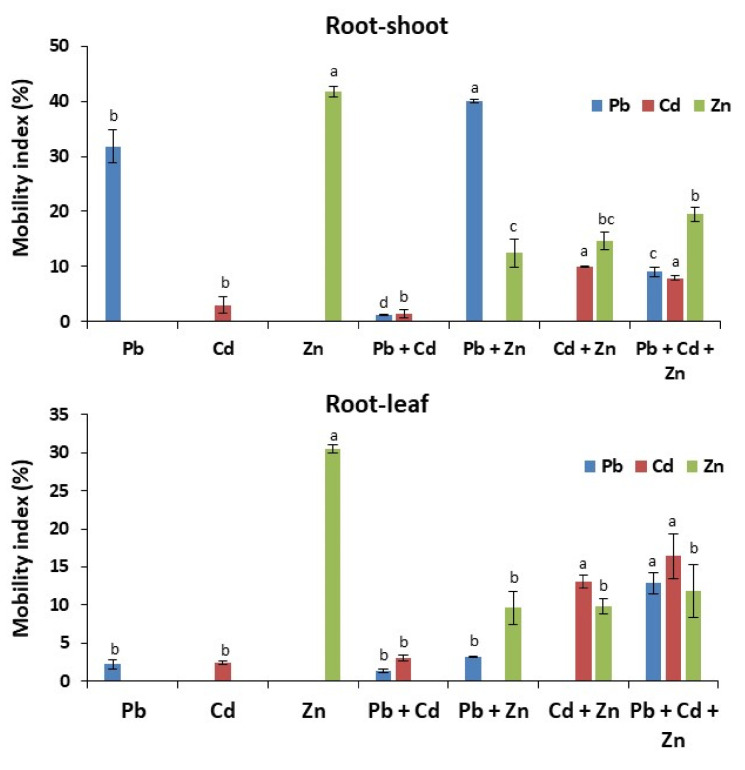
Mobility index (%) of Pb, Cd, and Zn from roots to shoots and roots to leaves in response to Pb/Cd/Zn applied alone and in combinations. The values marked with different letters are statistically different (*p* ≤ 0.05), while the values marked with the same letters do not differ significantly.

Interestingly, at the same concentration, plants preferentially accumulate Pb rather than Cd and Zn compared with control. Among Pb, Cd, and Zn treatments, only Zn concentration was significantly higher in leaves and shoots compared with control. This result indicates that Zn is more mobile to aerial parts of the maize plant than Pb and Cd (Table 1). Contrary to Zn, accumulated Cd was mainly blocked in roots, while accumulated Pb was transported in shoots as well. These observations were validated by calculating the mobility index (root to shoot and root to leaf). The mobility index classifies these tested metals as Zn > Pb > Cd, where Zn is the most mobile among the three metals (Figure 1). This indicates that, at the same concentration, each metal presents different accumulation and mobility to aerial parts.

We further analyzed if the combination of metals alters their accumulation or their transport throughout the plant. Therefore, we checked the metal accumulation in the different parts of the seedling after combined treatments, e.g., Pb + Cd, Pb + Zn, Cd + Zn, and Pb + Cd + Zn. To our surprise, the combinations of Pb with Zn or with both Zn and Cd, enhanced the total Pb accumulation in plants compared with the single Pb treatment (Table 1). Moreover, the addition of Zn enhanced the Pb transport to shoots and leaves compared with Pb treatment alone (Table 1). We also found that total Cd accumulation and transport to shoots and leaves was also enhanced in the presence of Zn. The increased mobility index of Pb/Cd after the addition of Zn further supports our observations (Figure 1). Contrary to Zn, the addition of Cd mainly blocked the Pb in the roots (Table 1 and Figure 1). Together, these results indicate that Zn favors the mobility of Pb and Cd to the aerial parts of the plants.

### 2.2. Pb/Cd Block the Accumulation and Transport of Zn to the Aerial Parts of Maize Plants

We then investigated whether the total accumulation and mobility of Zn are altered with the addition of Cd and Pb (Table 1 and Figure 1). We found that the total accumulation as well as the transport of Zn to shoots and leaves were decreased with the addition of Pb and/or Cd compared with the individual Zn treatment. Notably, Cd only facilitates the mobility of Pb in the presence of Zn but not alone. In brief, our results unravel the dynamic interaction of Pb, Cd, and Zn for their accumulation and transport to aerial parts.

### 2.3. Zn, Pb, and Cd Influence the Mobility of Divalent Calcium (Ca) and Magnesium (Mg) But Not the Monovalent Potassium (K)

We next investigated whether Pb, Cd, and Zn dynamics can influence the accumulation of these other divalent metals as well as their transport to aerial parts (Table 2). Total Ca and Mg accumulation was increased in the presence of Zn compared with control. However, in the presence of Cd, total Ca was decreased, while Mg was increased. The transport of Ca and Mg to aerial parts was differently affected in response to Pb, Cd, and Zn. For example, Ca and Mg were mainly blocked in the roots in the presence of Cd, whereas Ca was transported to shoots and leaves in the presence of Pb.

We further analyzed if the combination of Pb, Cd, and Zn alters the accumulation of Ca and Mg as well as their transport in aerial parts of the plants. We found that the levels of Ca and Mg in shoots and leaves were different between the Pb/Cd/Zn individual treatments and their combinations (Pb + Cd, Pb + Zn, Cd + Zn, and Pb + Cd + Zn). For example, in the presence of Pb + Zn, Ca and Mg accumulation in leaves was further increased compared with Zn treatment alone. These results show that Pb, Cd, and Zn dynamics influence the Ca and Mg mobility to aerial parts of the plants. However, we found that K levels did not change in response to Pb, Cd, and Zn treatments (Table S1), suggesting that Pb, Cd, and Zn dynamics are specific to divalent metals.

### 2.4. Antioxidant Activity Is Altered in Response to Zn, Pb, and Cd Applied Alone and in Combinations

**Figure 2:**
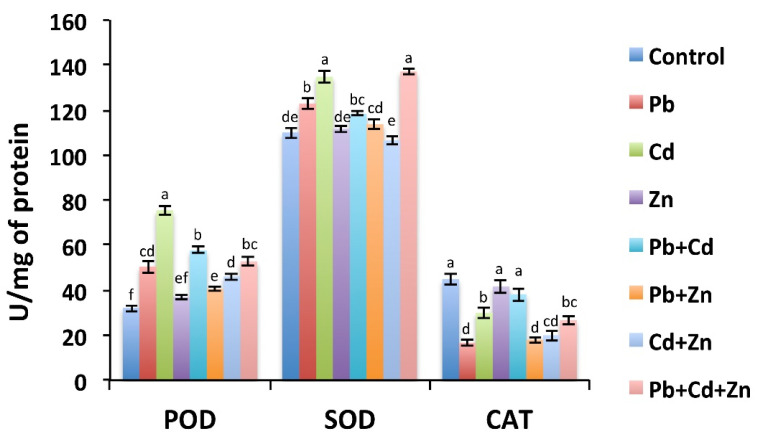
The activities of Peroxidase (POD), Superoxide dismutase (SOD), and Catalase (CAT) do not differ much between Pb/Cd/Zn applied alone and in combinations. The results shown are the average of three biological replicates. Bars represent mean ± SD. The values marked with different letters are statistically different (*p* ≤ 0.05), while the values marked with the same letters do not differ significantly.

Metal stress has been reported to alter the activity of Peroxidase (POD), Superoxide dismutase (SOD), and Catalase (CAT) antioxidant enzymes [39,40]. We then investigated whether the combinations of Zn, Pb, and Cd interfere with the POD, SOD, and CAT levels (Figure 2). The results show that POD and SOD activities were enhanced in response to Cd and Pb alone, while CAT activity was reduced compared with control. However, POD, SOD, and CAT activities did not change in response to Zn compared with control. POD and SOD levels were higher in response to the treatments with Pb + Cd and Zn + Pb + Cd compared with control. However, CAT level was decreased in response to Zn + Pb + Cd compared with control. Moreover, the addition of Zn with Pb or Cd resulted in a slight decrease of POD and SOD activities compared with individual Pb or Cd, while the levels of CAT were similar to individual Pb or Cd. Together, upon the combination of metals, the antioxidants activities were somehow similar to the individual metals.

### 2.5. Pb, Cd, and Zn Alone and in Combinations Differentially Regulate the Expression of ZIP Transporters

**Figure 3:**
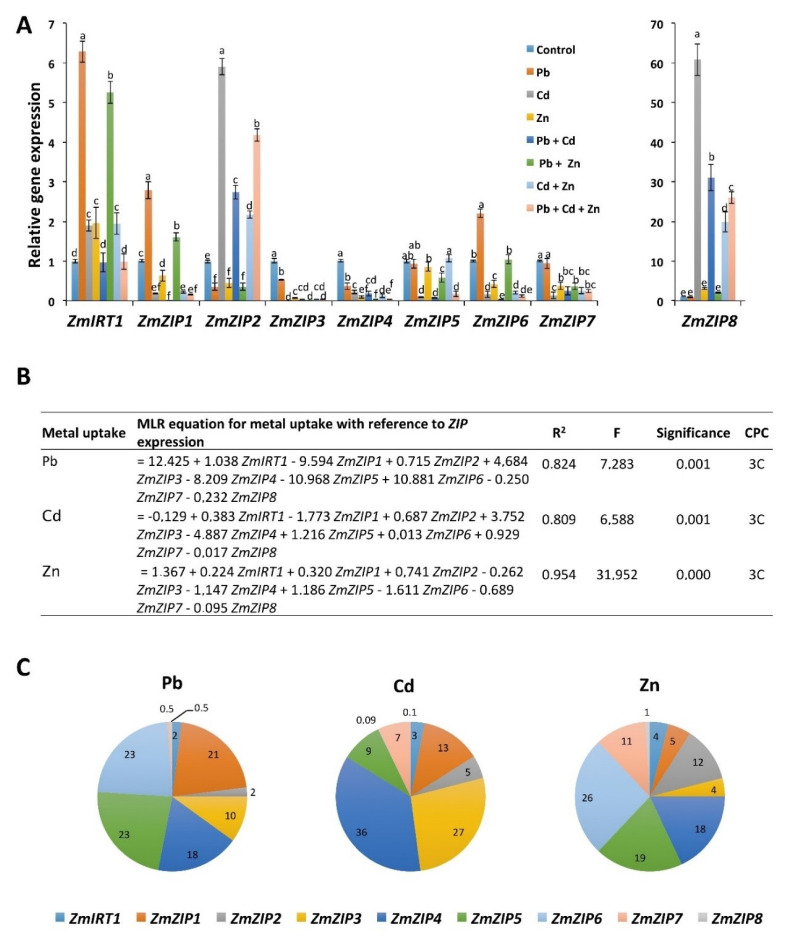
The expression of *ZIP* transporters in response to Pb/Cd/Zn applied alone and in combinations. (**A**) The expression of *ZIP* transporters was normalized with *ZmUBQ5*. The data presented are the averages of three biological replicates. Bars represent mean ± SD. The values marked with different letters are statistically different (*p* ≤ 0.05), while the values marked with the same letters do not differ significantly. (**B**) Multiple linear regression (MLR) equations for uptake of each heavy metal and expression of *ZIP* transporters. (**C**) Cumulative percentage contribution (CPC) for the expression of each *ZIP* transporter in response to the uptake of heavy metals.

The increased concentration of Pb/Cd in shoots and leaves with the addition of Zn led us to hypothesize that *ZIP* transporters may play a role in the transport of Pb/Cd to aerial parts of plants. We therefore investigated the expression of *ZIP* transporters in response to Zn, Pb, and Cd applied alone and in combinations. The results show that *ZIP* transporters present a differential expression in response to different metals, applied alone and in combinations (Figure 3). In response to Zn, *Iron-Regulated Transporter 1* (*IRT1*) expression was increased, whereas the expression of other *ZIP* transporters was decreased compared with control. In response to Pb treatment, the expression of *IRT1*, *ZIP1*, and *ZIP6* was increased compared with control, whereas the expression of *ZIP2*, *ZIP3*, and *ZIP4* was decreased compared with control. In response to Cd, the expression of *IRT1*, *ZIP2*, and *ZIP8* was increased compared with control, whereas the expression of other *ZIP* transporters was decreased compared with control. The expression of some genes was either increased or decreased in response to Pb, Cd, and Zn, but their levels were significantly different in many cases, e.g., *IRT1* expression was highest in response to Pb; *ZIP2* and *ZIP8* expressions were highest in response to Cd; and *ZIP1* and other *ZIP* gene expression was lower in response to Cd than that of Zn.

We next investigated whether the expression of *ZIPs* is altered when Pb, Cd, and Zn were combined. To our surprise, we found that the dynamics of *ZIPs* expression were changed in response to Pb + Cd, Pb + Zn, Cd + Zn, and Pb + Cd + Zn. In the case of Zn + Pb, the expression of *IRT1* and *ZIP1* was increased compared with control, but still lower than that of Pb alone. Furthermore, the expression of *ZIP6* did not change compared with control, while the expression of *ZIP2*, *ZIP3*, *ZIP4*, *ZIP5*, and *ZIP7* was similar to that of Zn alone. These results indicate that Zn encountered the increased expression of *IRT1*, *ZIP1*, and *ZIP6* compared with Pb alone. In the case of Cd + Zn, similar to Cd, the expression of *IRT1*, *ZIP2*, and *ZIP8* was increased compared with control, whereas the expression of other *ZIPs* was decreased. However, increased expression of *ZIP2* and *ZIP8* was lower than that of Cd alone, suggesting that Zn antagonizes the increased expression of *ZIP2* and *ZIP8* from Cd. In addition, the expression of other *ZIP* genes was at the similar level than that of Cd and/or Zn alone. In the case of Pb + Cd, the expression of *ZIP2* and *ZIP8* was increased compared with control, but their expression was lower than that of Cd treatment, suggesting that Pb antagonized the expression of *ZIP2* and *ZIP8* compared with Cd. However, the expression of other *ZIP* transporters was similar to that of Cd in Pb + Cd treatment. Furthermore, we combined all three metals and found that, similar to Cd and Zn + Cd, the expression of *IRT1*, *ZIP2*, and *ZIP8* was increased compared with control but still was lower than that of Cd, whereas the expression of other *ZIPs* was decreased to levels similar to Pb, Cd, and/or Zn levels. Together, these results show that, in addition to Zn, the expression of *ZIP* transporters is also regulated by Pb and Cd. Furthermore, metal combinations differently regulate the expression of *IRT1*, *ZIP1*, *ZIP2*, *ZIP6*, and *ZIP8* compared with individual metals.

The Multiple Linear Regressions (MLR) method was applied to find the linear relationship between explanatory variables (heavy metals uptake) and response variables (*ZIP* expression). The R^2^ value for the MLR equations between Pb, Cd, and Zn uptake models and *ZIPs* expression were 0.82, 0.80, and 0.95, respectively. This indicates a good fit of the model for all cases (Figure 3B). The highest percentage contribution in Pb uptake was with *ZIP5* and *ZIP6* (23%); for Cd with *ZIP4* (36%); and for Zn with *ZIP6* (26%) (Figure 3C). This further suggests the specific regulation of *ZIP* transporters in response to Pb, Cd, and Zn.

### 2.6. Pb, Cd, and Zn Alone and in Combinations Differentially Regulate the Expression of Histone Deacetylases (HDACs)

**Figure 4:**
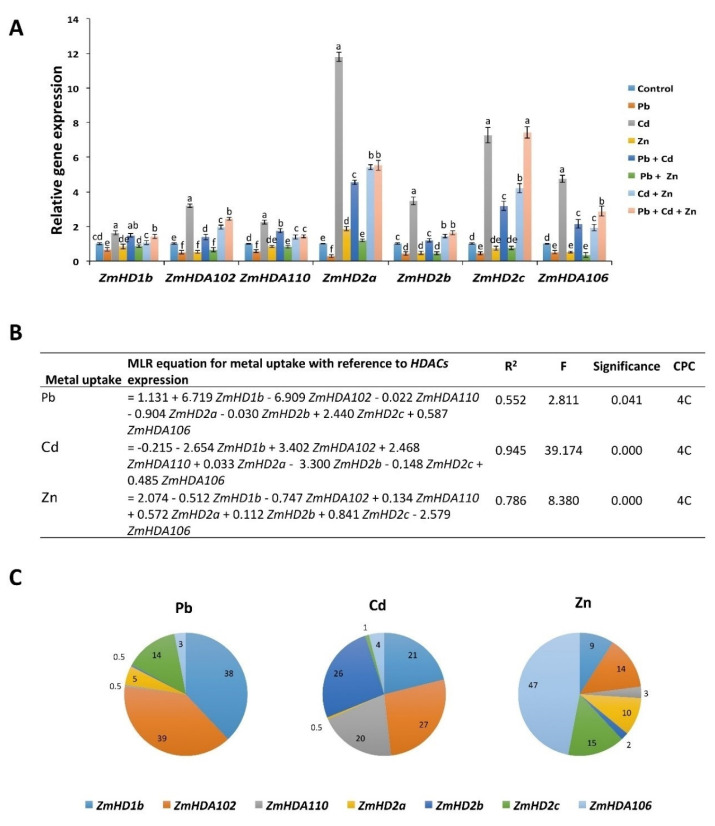
The expression of histone deacetylases in response to Pb/Cd/Zn applied alone and in combinations. (**A**) The expression of histone deacetylases was normalized with *ZmUBQ5*. The data presented are the averages of three biological replicates. Bars represent mean ± SD. The values marked with different letters are statistically different (*p* ≤ 0.05), while the values marked with the same letters do not differ significantly. (**B**) Multiple linear regression (MLR) equations for uptake of each heavy metal and expression of *HDACs*. (**C**) Cumulative percentage contribution (CPC) for expression of each *HDAC* transporter in response to the uptake of heavy metals.

Because heavy metals were reported to cause DNA hypo-acetylation in human [19], we hypothesized that *HDACs* could also play a role in plant adaptation under these metal stresses. Therefore, we checked the expression of all *HDACs* in maize in response to Pb, Cd, or Zn applied alone and in combination (Figure 4). We found that all *HDACs* respond differently to each metal. In response to Zn, the expression of *HDA102*, *HD2b*, *HD2c*, and *HDA106* was decreased compared with control, whereas the expression of *HD2a* was increased. In response to Pb, the expression of all *HDACs* was downregulated compared with control. On the contrary, in response to Cd, the expression of all the *HDACs* was increased compared with control and the increase in expression of *HD2a* was even more than that of Zn alone. These results indicate the different regulation of *HDACs* in response to Pb, Cd, and Zn, as well as suggest different acetylation levels. For example, Pb and Cd could result in histone hyperaectylation and histone hypoacetylation, respectively.

We next investigated the expression of *HDACs* in response to the combination of Pb, Cd, and Zn (Figure 4). In response to Cd + Zn, the expression of *HDA102*, *HDA110*, *HD2a*, *HD2b*, *HD2c*, and *HDA106* was increased compared with control, while their expression was still lower than that of Cd alone. In response to Cd + Pb, the expression of *HD1b*, *HDA102*, *HDA110*, *HD2a*, *HD2c*, and *HDA106* was increased compared with control but still lower than that of Cd alone. This indicates that Zn and Pb antagonize the Cd-mediated increase in the expression of all *HDACs*. In response to Pb + Zn, the expression of *HDA102*, *HD2b*, *HD2c*, and *HDA106* decreased compared with control, to similar levels as for Pb or Zn alone. However, we did not detect the expression of the histone deacetylases *RPD3*, *HDA1*, and *HDA108* in maize roots treated with Pb, Cd, and Zn, either alone or in combination.

We next applied the MLR equation on heavy metal uptake and *HDACs* expression. The R^2^ value for the MLR equations between Pb, Cd, and Zn uptake models and *HDACs* expression were 0.55, 0.94, and 0.78, respectively. This indicates a good fit of the model for all cases (Figure 4B). The highest percentage contribution in Pb uptake was with *HDA102* (39%); for Cd with *HDA102* (27%); and for Zn with *HDA106* (47%) (Figure 4C). This further suggests the specific regulation of *HDACs* in response to Pb, Cd, and Zn.

### 2.7. Pb, Cd, and Zn Alone and in Combinations Differentially Regulate the Expression of DNA Methyltransferases

**Figure 5:**
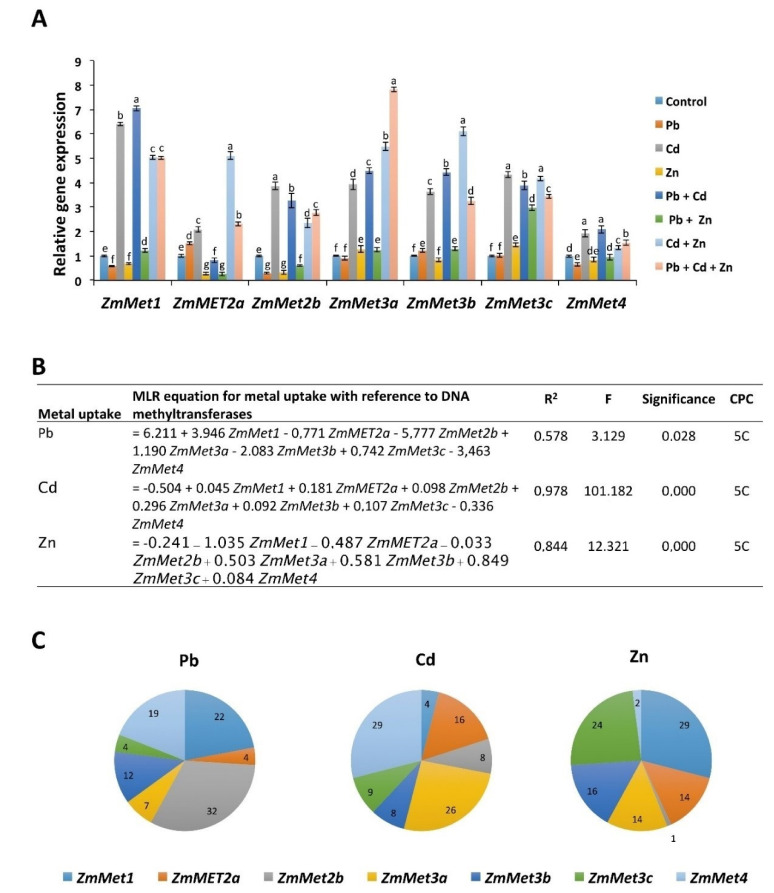
The expression of DNA methyltransferases in response to Pb/Cd/Zn applied alone and in combinations. (A) The expression of DNA methyltransferases was normalized with *ZmUBQ5*. The data presented are the averages of three biological replicates. Bars represent mean ± SD. The values marked with different letters are statistically different (*p* ≤ 0.05), while the values marked with the same letters do not differ significantly. (**B**) Multiple linear regression (MLR) equations for uptake of each heavy metal and expression of DNA methyltransferases. (**C**) Cumulative percentage contribution (CPC) for expression of each DNA methyltransferase in response to the uptake of heavy metals.

Pb, Cd, and Zn phytotoxicity has been shown to alter the DNA methylation levels at metal transporters to confer the metal tolerance in wheat [28]. We hypothesized that Pb, Cd, and Zn alone and in combinations could alter the DNA methylation levels at the promoter of *ZIP* transporters through the regulation of DNA methyltransferases in maize. Therefore, we checked the expression of maize DNA methyltransferases [24]. The results show that in response to Zn the expression of *Methyltransferase 1 (MET1*), *MET2a*, and *MET2b* was decreased compared with control, whereas the expression of *MET3a* and *MET3c* was increased (Figure 5). In response to Pb, the expression of *MET1*, *MET2b* and *MET4* was downregulated compared with control, whereas the expression of *MET2a* and *MET3b* was increased. Contrary to Pb and Zn, the expression of all the DNA methyltransferases was increased in response to Cd. Although the expression of DNA methyltransferases was increased with Pb/Cd/Zn, their levels were still significantly different among Pb, Cd, and Zn. For example, *MET2a* and *MET3b* expression levels were the highest in response to Cd compared with Pb. In response to Cd, the expression of *MET3a* and *MET3c* was more than that of Zn, which indicates the different regulation of DNA methyltransferases depending on the metal stress.

We also evaluated the expression of DNA methyltransferases in combination of metals (Figure 5). In response to Cd + Zn, the expression of all the methyltransferases was increased compared with control. However, the expression of *MET1*, *MET2b*, and *MET4* was lower than that of Cd, and the expression of *MET2a*, *Met3a* and *MET3b* was even higher than that of Cd. In response to Pb + Cd, the expression of all the DNA methyltransferases was higher than that of control, except for *MET2a*. Furthermore, the expression of *MET1*, *MET3a*, and *MET3b* was higher than that of Cd and the expression of *MET2b* and *MET3c* was lower than that of Cd. However, the expression of *MET4* was similar to Cd. In response to Pb + Zn, *MET3c* showed a strong increase in the expression compared with control and Pb/Zn. In response to Pb + Cd + Zn, the expression of all DNA methyltransferases was increased compared with control but the expression was either similar to Zn + Cd or Cd alone. Interestingly, the expression of *MET3a* was higher than that of Cd or Zn + Cd. Together, the expression levels of DNA methyltransferases are different among Pb, Cd, and Zn individual treatments and their combinations.

We next applied the MLR equation on heavy metal uptake and DNA methyltransferases expression. The R^2^ value for the MLR equations between Pb, Cd, and Zn uptake models and *METs* expression were 0.57, 0.97, and 0.84, respectively. This indicates a good fit of the model for all cases (Figure 5B). The highest percentage contribution in Pb uptake was with *Met2b* (32%); for Cd with *Met4* (29%); and for Zn with *Met1* (29%) (Figure 5C). This further suggests the specific regulation of DNA methyltransferases in response to Pb, Cd, and Zn.

### 2.8. Zn, Pb, and Cd Combinations Alter the DNA Methylation Levels at the Promoter of ZIP Transporters

**Figure 6:**
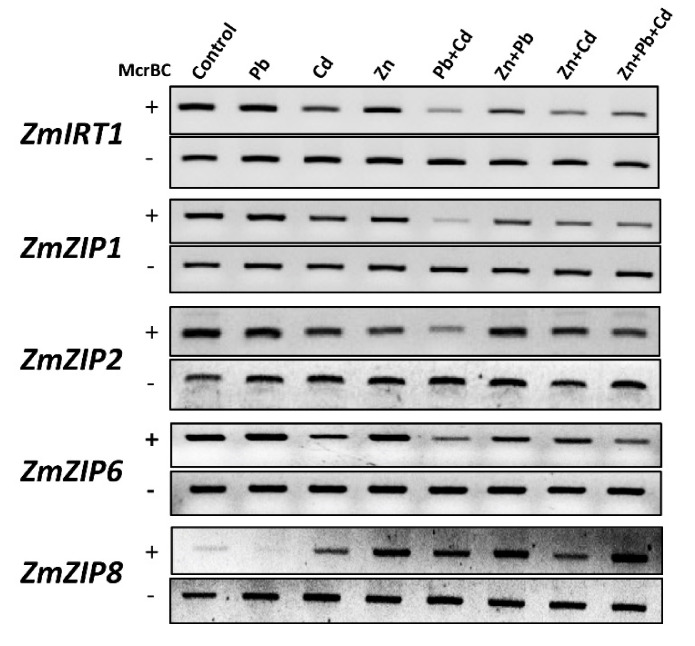
DNA methylation levels at the promoter of selected *ZIP* transporters in response to Pb/Cd/Zn applied alone and in combinations. DNA was digested with McrBc and equal amounts of digested or undigested DNA were used as template for PCR. McrBC digests the methylated DNA; therefore, lighter band intensity reflects higher DNA methylation level.

Because Pb, Cd, and Zn alone and in combination differently regulate the expression of DNA methyltransferases (Figure 5), we therefore investigated the DNA methylation levels at the promoter of *ZIP* transporters. We employed Chop-PCR to detect the non-methylated DNA levels at the promoter of some selected candidates, i.e., *IRT1*, *ZIP1*, *ZIP2*, *ZIP6*, and *ZIP8* (Figure 6 and Figure S1). We used these *ZIP* transporters because their gene expression was more dynamic in response to the combination of Pb, Cd, and Zn (Figure 3). Consistent with the upregulation of all the DNA methyltransferases in response to Cd, DNA methylation levels were increased at *IRT1*, *ZIP1*, *ZIP2*, and *ZIP6* compared with the control (Figure 6). However, DNA methylation at *ZIP8* was decreased compared to the control in response to Cd. In response to Pb, DNA methylation was increased at *ZIP2* and *ZIP8* compared to control. In response to Zn, DNA methylation levels were also increased at *IRT1*, *ZIP1*, *ZIP2*, and *ZIP6* compared to control, but decreased at *ZIP8*. These results indicate that each metal leads to distinct DNA methylation levels at specific loci.

In general, the expression of all the DNA methyltransferases was increased in response to the combination of metals compared with Cd alone, e.g., Pb + Cd, Cd + Zn, and Pb + Cd + Zn (Figure 5). Consistent with this observation, in response to Pb + Cd, the methylation levels were further increased at *IRT1*, *ZIP1*, *ZIP2*, and *ZIP6* compared to Cd, but decreased at *ZIP8* (Figure 6). In response to Cd + Zn and Pb + Cd + Zn, DNA methylation levels were also increased at *IRT1*, *ZIP1*, and *ZIP6* compared with control as well as Zn and Pb. These results indicate that Pb, Cd, and Zn interaction further alters the DNA methylation levels at specific loci compared with individual metals.

**Figure 7:**
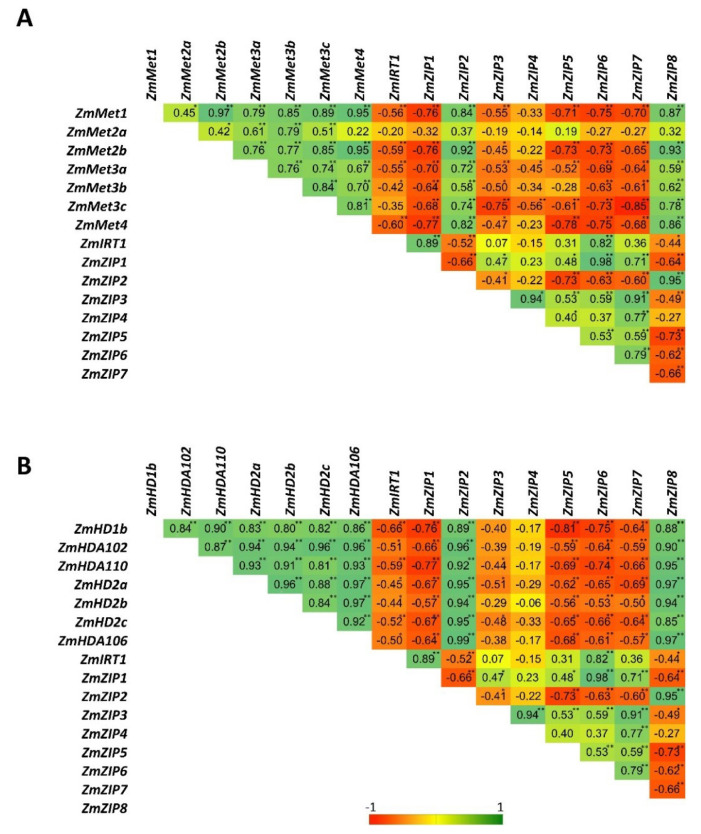
Pearson correlation between the expression of *ZIP* transporters and the expression of DNA methyltransferases (**A**) and *HDACs* (**B**). Red color represents the negative correlation (−1), green color represents the positive correlation (+1), and yellow represents the absence of correlation (0). * significant correlation at *p* ≤ 0.05, ** significant correlation at *p* ≤ 0.01.

Pearson’s correlation coefficient (r) is a measure of the strength of the association between two variables. We therefore calculated the Pearson correlation between the expression of *ZIPs* and the expression of *HDACs*/DNA methyltransferases (Figure 7). The expression of *IRT1*, *ZIP1*, *ZIP3*, *ZIP5*, *ZIP6*, and *ZIP7* showed negative correlation with the expression of *HDACs* and DNA methyltransferases. These results suggest that *HDACs* and DNA methyltransferases together negatively regulate the expression of some *ZIPs*. Furthermore, the expression of *ZIP2* and *ZIP8* showed a positive correlation with the expression of *HDACs* and DNA methyltransferases, suggesting that the regulation of *ZIP2* and *ZIP8* could be independent of *HDACs* and DNA methyltransferases.

## 3. Discussion

In response to fluctuating environment, chromatin landscape is achieved through post-transcriptional histone modifications to regulate the gene expression. Here, we studied the dynamic metal interactions among Zn, Pb, and Cd in maize and found that the presence of Zn facilitates the accumulation and transport of Pb and Cd in the aerial parts of the maize plants. Furthermore, we showed that Pb, Cd, and Zn alone and in combinations alter the expression of DNA methyltransferases, which consequently change the DNA methylation levels at the promoter of some *ZIP* transporters to regulate their expression.

Arabidopsis MET1 (mammalian DNA Methyltransferase 1 (Dnmt1)) and plant specific Chromomethylases (CMTs) are reported to maintain the CG and CHG methylation, respectively [41,42]. Moreover, Arabidopsis Domains Rearranged Methylases (DRMs) (mammalian Dnmt3) are reported to establish all the methylation contexts, especially CHH methylation [43]. ZmMET1 is the homolog of MET1 and Dnmt1; ZmMET2a and ZmMET2b are the homologs of plant specific Arabidopsis CMTs; and ZmMET3a, ZmMET3b, and ZmMET3c are homologs of Arabidopsis DRMs and mammalian Dnmt3 de novo DNA methyltransferases. Our results show that Pb, Cd, and Zn alone and in combination regulate the expression of maize DNA methyltransferases. Furthermore, MLR analysis between the expression of DNA methyltransferases and uptake of Pb/Cd/Zn showed that the highest percentage of expected contribution in Pb uptake was with *Met2b* and *Met1* (32% and 22%, respectively); for Cd with *Met4* and *Met3a* (29% and 26%, respectively); and for Zn with *Met1* and *Met3c* (29% and 24%, respectively). This indicates the specific regulation of each DNA methyltransferase in response to Pb, Cd, and Zn. It also indicates that one DNA methyltransferase could be regulated by more than one metal. In response to metal stress, changes in DNA methylation have been reported in wheat, *Vicia faba*, rape seedlings, white clover, industrial hemp hyper-accumulators plants, and *Arabidopsis* [25,26,28,44,45,46]. Interestingly, DNA methylation changes in response to Cd stress depend on the plants, e.g., DNA hypomethylation in rape seedling, white clover and industrial hemp [25,26], while DNA hypermethylation in *Vicia faba* [46]. Our data show that the expression of all DNA methyltransferases is increased in response to Cd treatment, suggesting DNA hypermethylation in response to Cd in maize. Indeed, DNA methylation levels at *IRT1*, *ZIP1*, *ZIP2*, and *ZIP6* confirm the DNA hypermethylation at these loci in response to Cd. Furthermore, DNA methylation levels were also slightly increased in response to Zn or Pb at specific *ZIP* transporters. These results indicate the specific DNA methylation changes in response to Pb, Cd, and Zn. Notably, we also observed further increase in DNA methylation levels at *ZIP* transporters in response to the combination of metals, e.g., DNA methylation levels at *IRT1*, *ZIP1*, *ZIP2*, and *ZIP6* in response to Pb + Cd. This indicates that the combination of metals could produce different DNA methylation levels compared with individual metals. However, further studies are required to investigate the genome wide DNA methylation status as well as targets of DNA methyltransferases in response to metals applied individually or in combination.

Similar to DNA methylation, the expression of all *HDACs* also responds differently to the individual metals as well as the combination of metals. Furthermore, MLR analysis also suggests the specific regulation of *HDACs* in response to Pb, Cd, and Zn uptake. These observations suggest that Pb, Cd, and Zn could cause different histone acetylation profiles. Interestingly, our Pearson correlation analysis showed a negative correlation between the expression of *HDACs*/DNA methyltransferases and the expression of *IRT1*, *ZIP1*, *ZIP3*, *ZIP5*, *ZIP6*, and *ZIP7*. This suggests that altered DNA methylation and histone acetylation levels, alone or together, could regulate the *ZIPs* gene expression. However, the expression of *ZIP2* and *ZIP8* showed a positive correlation with the expression of *HDACs* and DNA methyltransferases, suggesting that the regulation of these *ZIP* transporters could be independent of *HDACs* and DNA methyltransferases. However, further studies are required to validate the crosstalk between histone acetylation and DNA methylation to regulate the *ZIPs* gene expression.

**Figure 8:**
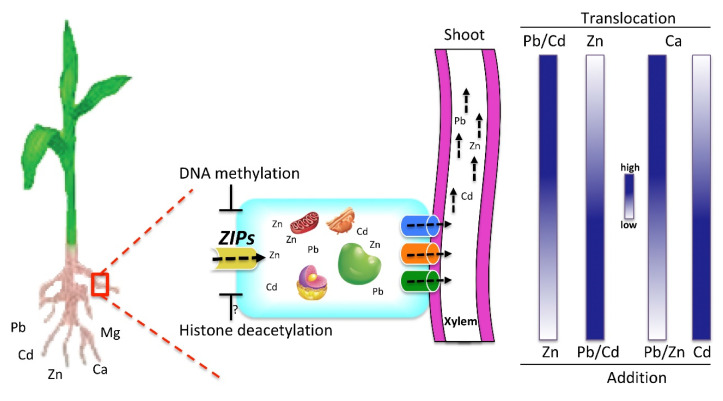
Proposed model for the uptake and translocation interplay of Pb, Cd, and Zn through *ZIP* transporters. Heavy metals may alter the DNA methylation and histone acetylation levels through the activity of DNA methyltransferases and histone deacetylases, respectively. The resulting chromatin landscape may control the expression of certain *ZIP* transporters that may carry Pb and Cd along with Zn into the cell. Consequently, other metal transporters may facilitate the loading of toxic metals to xylem, and subsequent transport to shoots that result in the increased concentration of toxic metals in aerial parts of the plants. Moreover, the enrichment of toxic metals into the cell disturbs the uptake and translocation of other essential divalent metals, e.g., Ca/Mg. The gradient represents the metal concentration, and cylindrical structures represent the metal transporters that can load the metals to the xylem.

Our results also show an interesting plant preference of metal accumulation and mobility to different parts of the plants. To our surprise, the addition of Zn facilitates the accumulation of Pb and Cd to the aerial parts of the plants. Furthermore, Pb, Cd, and Zn dynamics seem specific to divalent metals, as K levels did not change after treatments. There are two possible explanations for this phenomenon. Firstly, Pb and Cd could be potentially co-transported with Zn. Secondly, Pb and Cd could allosterically regulate the ability of some *ZIP* transporters to transport Pb and Cd. Notably, we also observed that Pb and Cd also regulate the expression of *ZIP* transporters, e.g., *IRT1*, *ZIP1*, and *ZIP6* expression increased in response to Pb, while the expression of *ZIP2*, *ZIP3*, and *ZIP4* decreased compared with control. Furthermore, MLR analysis also suggests that Pb, Cd, and Zn specifically regulate the expression of certain *ZIP* transporters. Interestingly, the *ZIP* family has been reported to uptake Fe, Mn, and Cd [13,16,17,47] in other plants, questioning the specificity of *ZIP* metal transporters. Moreover, Arabidopsis IRT1, a *ZIP* transporter, has also been reported to transport multiple metals, including Fe, Zn, Mn, and Cd [48,49]. These observations suggest that maize *ZIP* transporters may also transport Pb/Cd. Therefore; we propose that heavy metals alter the DNA methylation and histone acetylation levels through the activity of DNA methyltransferases and histone deacetylases (Figure 8). The resulting chromatin landscape may control the expression of certain *ZIP* transporters that may carry Pb and Cd along with Zn into the cell. Furthermore, the accumulation of Pb and/or Cd will disturb the cellular homeostasis of other essential divalent metals. Consequently, maize plant tries to antagonize the metal toxicity through the activity of SOD, POD, and CAT in response to Pb, Cd, and Zn. However, upon the combination of metals, the antioxidants activities were somehow similar to the individual metals. This suggests that maximum activity of CAT, SOD, and POD was already achieved in response to individual metals. Therefore, the combination of metals would not further elevate their response. Eventually, the excess of these toxic metals might then be loaded to xylem, and transported to aerial parts through the activity of multiple transporters, for example Heavy Metal ATPases (HMAs), Yellow Stripe-Likes (YSLs), etc. [50,51,52]. In this scenario, achieving the specificity of *ZIP* metal transporters that specifically uptake Zn and block Pb and Cd is desired and need further investigations. Zn is being applied as a fertilizer. In the light of our results, if the agriculture land is already polluted with divalent metals, the application of Zn could make toxic metals more mobile to aerial parts of plants. Furthermore, the presence of Pb/Cd in the soil can block the Zn accumulation and transport to aerial parts of the plants. However, how plant imports preferentially one metal over the other metals, and how the metal combinations change metal accumulation and mobility dynamic need further investigations.

## 4. Materials and Methods

### 4.1. Hydroponics

Maize seeds (*Zea Mays* L. cv. NK-8441 Syngenta) were placed on a filter paper humidified with 0.5 mM solution of CaSO_4_ and placed in incubator at 28 °C for 48 h to germinate. After germination, two-day-old young seedlings of similar size were transferred to hydroponics medium containing 0.2 mM KH_2_PO_4_, 1 mM K_2_SO_4_, 2 mM Ca(NO_3_)_2_, 2 mM CaCl_2_, 0.5 mM MgSO_,_ 0.2 mM FeSO_4_ . 7H_2_O, 5 µM H_3_BO_3_, 2 µM MnSO_4_, 0.5 µM ZnSO_4_, 0.3 µM CuSO_4_, and 0.01 µM (NH_4_)_2_Mo_7_O_24_. Hydroponics medium was changed twice a week. The heavy metal treatment was applied at three leaf stage by adding Cd (100 µM), Pb (100 µM), and Zn (100 µM) alone or in combination using completely randomized design (CRD). Cd, Pb, and Zn were added as CdCl_2_, Pb(NO_3_)_2_, and ZnSO_4_, respectively, and their concentrations were selected based on previously published reports [53,54,55]. The seedlings were grown in a greenhouse under an average day/night temperature of 28/18 °C, a photoperiod of 14 h light (≥350 μmol m^−2^ s^−1^ PAR)/10 h dark, and a relative humidity of 65% ± 5%.

### 4.2. Atomic Absorption Analysis

Two weeks after treatment with Pb, Cd, or Zn, freshly harvested maize plants were washed with double distilled water (distilled water passed through Millipore filters) to remove any soil particles and air-borne pollutants. The plants were divided into roots, shoots, and the whole 4th leaf for atomic absorption analysis. After taking their fresh weight, the samples were placed in an oven at 60 °C for 72 h until a constant dry weight was attained using weight balance (Panther-USA, model-BM-32). Plant materials were ground (<0.2 mm) and about 150 mg of dried plant material were used for the analysis of Pb, Cd, and Zn. The material was burned into ashes at 550 °C for 5 h in the furnace and digested with 2 mL of 4 M HNO_3_ for 3 h. Subsequently, double distilled water (≈8 mL) was used to make a final volume of 10 mL. The digested plant material from different parts of the plants was filtered (i.e., using Whatman filter paper No. 21) and analyzed for Pb, Cd, and Zn concentrations through the atomic absorption spectrophotometer (Perklin Elmer AAnalyst 700), as suggested by A.O.C.A (association of official analytical chemist) protocol [56]. The instrument was calibrated with calibration blank and three series of working standard solutions of studied metals. Atomic absorption spectroscopy standards of Merck for each analysed metal were used in this study.

### 4.3. Extraction and Quantification of Antioxidant Enzymes

Green leaf samples were harvested from plants and quickly immersed in liquid nitrogen. The frozen samples (0.5 g) were homogenized in freshly prepared potassium phosphate buffer (100 mM potassium phosphate pH 7 and 0.1 mM EDTA). After centrifugation at 4 °C and 13,000× *g* for 5 min, the supernatant was collected in eppendorf tubes and used for the analysis of antioxidant enzymes. Catalase, peroxidase and superoxide dismutase activities were determined as described previously [57]. The CAT activity was determined by monitoring the decomposition of H_2_O_2_ at 240 nm through spectrophotometer (U2020 IRMECO, Germany), while the activity of POD was measured by using guaiacol as substrate. The reaction mixture contained 0.1 M phosphate buffer of pH 7, 1% guaiacol, 0.4 M H_2_O_2_, and the enzyme extract. Change in absorbance per unit time was measured at 470 nm. SOD activity was measured by photoreduction of nitroblue tetrazolium (NBT). Reaction mixture comprised 50 mM phosphate bufer of pH 7.8, 0.1 mM EDTA, 20 mM L-methionine, 750 µM NBT, 20 µM riboflavin, and the enzyme extract. The amount of protein was also determined according to the protocol of Lowry [58].

### 4.4. Gene Expression Analysis

After 48 h of treatment with Pb, Cd, and Zn applied alone and in combinations, total RNA from roots was extracted by using Trizol (Invitrogen, Carlsbad, CA, USA) according to the manufacturer’s instructions. DNase I treatment was performed with DNAse (Takara, Shiga, Japan), and reverse transcription was performed with Superscript III (Invitrogen, Carlsbad, CA, USA) using the gene-specific primers as described previously [59]. RT-qPCR was performed with the gene-specific primers using SYBR green Master Mix (Roche, Basel, Switzerland) as described in [59]. Maize Ubiquitin *5* (*ZmUBQ5*) was used as an internal reference gene to normalize the data. Primer sequences for qRT-PCR are listed in Table S2.

### 4.5. ChoP-PCR

Chop-PCR was performed as previously described [60]. Briefly, genomic DNA was extracted with CTAB from roots after 48 h of treatment with Pb, Cd, and Zn applied alone and in combinations. Then, the DNA was digested with a methylation-sensitive restriction enzyme (McrBc). Equal amounts of digested and undigested DNA were used as templates for 32 cycles of PCR amplification, followed by agarose gel electrophoresis and ethidium bromide staining. Chop-PCR primers are listed in Table S2.

### 4.6. Statistical Analysis

Each treatment was applied in three biological replicates and there were three technical replicates in each biological replicate regarding the biochemical analysis. Data of means from each biological replicates were subjected to theGeneral Linear Model (GLM) to know the effect of treatments on each studied parameter. Furthermore, after doing analysis of variance (ANOVA), post hoc multiple comparison (least significant difference test) was performed to compare and rank the treatments means value by using alphabets. Statistical analyses were performed at significance level of *p* ≤ 0.05. In the next step, correlation between studied parameters was found by using Pearson Correlation. Multiple linear regressions (MLR) analysis was constructed between dependent variables (heavy metal uptake) and predictors (*ZIP* expression, DNA methyltransferases and *HDACs* expression). The MLR equation was used as described below [61]

(1) $$Y=b_{0}\;+\;b_{1}x_{1}\;+\;b_{2}x_{2}\;+\;\ldots \;+\;b_{\mathrm{xth}}\;x_{\mathrm{xth}}$$

Y is the predicted value of the dependent variable (heavy metal uptake), b_0_ is the value of multiple regression constant, b_1-xth_ is the value of regression constant of studied parameters, and x_1–xth_ represents the studied variables of *ZIP* expression, DNA methyltransferases, and *HDACs* expression.

The R^2^ value represents the coefficient of multiple linear regression and it denotes worthwhile competence between predicted and quantified values. A higher R^2^, more than 0.75, is considered a good fit model of MLR for metal uptake and studied parameters. Cumulative percentage contributions (CPC) represents the contribution of studied parameters against heavy metal uptake and calculated by using the following equation;

(2) $$B=\frac{\mathrm{bi}}{\sum \mathrm{bi}}\times 100$$

B is the cumulative percentage contribution of studied parameter, bi is the value of MLR coefficients of each studied parameter, and Ʃbi is the value of sum of MLR coefficients of all studied parameters.

Mobility Index was calculated by using the following formula;

(3) $$\mathrm{Mobility Index}\;\left(\%\right)\;=\frac{\mathrm{Concentration of metal in the receiving level}}{\mathrm{Concentration of metal in the source level}}\times 100$$

All the statistical analyses were performed using IBM SPSS statistical software.

## 5. Conclusions

This study show that Zn, Pb, and Cd interfere with each other’s accumulation and transport to aerial parts of the maize plants. Further, the interplay among Zn, Pb, and Cd specifically alters the uptake and translocation of divalent calcium and magnesium but not the monovalent potassium. Finally, we showed that DNA methyltransferases and histone deacetylases together regulate the expression of *ZIP* transporters, which are most likely implicated in the transport of Pb, Cd, and Zn. This study demonstrates that epigenetic regulators may play an important role against the tolerance of heavy metals in maize. However, further studies, such as on genome wide DNA methylation changes and histone acetylation changes in response to metal stress alone and in combination, will advance our understandings regarding their role in the tolerance to metal stress.

## Figures and Tables

**Table 1:** Pb, Cd, and Zn levels in roots, shoots, and leaves in response to Pb/Cd/Zn alone or in combinations.

Treatments	Pb (mg/g)				Cd (mg/g)				Zn (mg/g)			
	Leaf	Shoot	Root	Total	Leaf	Shoot	Root	Total	Leaf	Shoot	Root	Total
Control	0.10 ^c^	0.08 ^d^	0.08 ^d^	0.27 ^c^	0.01 ^c^	0.01 ^d^	0.01 ^c^	0.03 ^c^	0.10 ^cd^	0.03 ^d^	0.08 ^d^	0.22 ^d^
Pb	0.12 ^c^	1.78 ^b^	5.62 ^c^	7.52 ^b^	0.01 ^c^	0.01 ^d^	0.02 ^c^	0.04 ^c^	0.03 ^d^	0.04 ^d^	0.06 ^d^	0.14 ^d^
Cd	0.11 ^c^	0.22 ^d^	0.09 ^d^	0.42 ^c^	0.04 ^c^	0.05 ^c^	1.92 ^b^	2.02 ^b^	0.06 ^d^	0.04 ^d^	0.07 ^d^	0.19 ^d^
Zn	0.09 ^c^	0.06 ^d^	0.10 ^d^	0.26 ^c^	0.03 ^c^	0.01 ^d^	0.02 ^c^	0.06 ^c^	0.67 ^a^	0.92 ^a^	2.20 ^a^	3.80 ^a^
Pb + Cd	0.09 ^c^	0.09 ^d^	7.23 ^b^	7.43 ^b^	0.05 ^c^	0.02 ^cd^	1.68 ^b^	1.75 ^b^	0.03 ^d^	0.03 ^d^	0.11 ^d^	0.18 ^d^
Pb + Zn	0.22 ^b^	2.76 ^a^	6.90 ^b^	9.89 ^a^	0.04 ^c^	0.03 ^cd^	0.11 ^c^	0.19 ^c^	0.19 ^bc^	0.24 ^c^	1.98 ^c^	2.41 ^c^
Cd + Zn	0.13 ^c^	0.14 ^d^	0.17 ^d^	0.99 ^c^	0.40 ^b^	0.30 ^a^	3.07 ^a^	3.78 ^a^	0.20 ^bc^	0.30 ^c^	2.06 ^b^	2.57 ^bc^
Pb + Cd + Zn	1.10 ^a^	0.77 ^c^	8.60 ^a^	10.49 ^a^	0.47 ^a^	0.22 ^b^	2.88 ^a^	3.58 ^a^	0.25 ^b^	0.41 ^b^	2.11 ^ab^	2.78 ^b^

Plants were grown in hydroponic culture, and metal accumulation was investigated after two weeks of treatment. The results shown are the average of three biological replicates. The values marked with different letters are statistically different (*p* ≤ 0.05), while the values marked with the same letters do not differ significantly. The statistical analyses were performed to examine the changes in each plant fraction i.e., shoots, roots, or leaves with applied treatments. Statistical analyses between different plant fractions are not given in the table.

**Table 2:** Calcium and magnesium levels in roots, shoots, and leaves in response to Pb/Cd/Zn alone or in combinations.

Treatments	Ca (mg/g)				Mg (mg/g)			
	Leaf	Shoot	Root	Total	Leaf	Shoot	Root	Total
Control	20.76 ^c^	17.08 ^b^	25.45 ^b^	63.29 ^bc^	5.175 ^ab^	3.22 ^cd^	2.55 ^e^	10.96 ^de^
Pb	21.78 ^bc^	27.59 ^a^	10.68 ^e^	60.06 ^c^	3.47 ^d^	5.53 ^a^	3.57 ^d^	12.57 ^cd^
Cd	12.03 ^d^	8.99 ^c^	20.69^bcd^	41.72 ^d^	4.301 ^bcd^	6.07 ^a^	5.46 ^ab^	15.84 ^ab^
Zn	26.29 ^b^	26.29 ^a^	39.34 ^a^	91.93 ^a^	4.98 ^ab^	4.82 ^ab^	5.67 ^ab^	15.48 ^ab^
Pb + Cd	15.20 ^d^	9.28 ^c^	21.89 ^bc^	46.38 ^d^	3.78 ^cd^	2 ^d^	4.25 ^cd^	10.04 ^e^
Pb + Zn	36.52 ^a^	26.90 ^a^	25.77 ^b^	89.21 ^a^	5.56 ^a^	4.03 ^bc^	4.90 ^bc^	14.51 ^bc^
Cd + Zn	21.05 ^c^	29.07 ^a^	19.24 ^cd^	69.37 ^b^	4.54 ^bc^	5.40 ^a^	5.47 ^ab^	15.42 ^ab^
Pb + Cd + Zn	26.62 ^b^	27.86 ^a^	16.32 ^d^	70.81 ^b^	5.56 ^a^	5.46 ^a^	5.98 ^a^	17.01 ^a^

Plants were grown in hydroponic culture, and metal accumulation was investigated after two weeks of treatment. The results shown are the averages of three biological replicates. The values marked with different letters are statistically different (*p* ≤ 0.05), while the values marked with the same letters do not differ significantly. The statistical analyses were performed to examine the changes in each plant fraction i.e., shoots, roots, or leaves with applied treatments. Statistical analyses between different plant fractions are not given in the tables.

## Supplementary Materials

Supplementary materials can be found at https://www.mdpi.com/1422-0067/21/18/6959/s1.
