# Supplementary material for: The Interplay between Toxic and Essential Metals for Their Uptake and Translocation Is Likely Governed by DNA Methylation and Histone Deacetylation in Maize
Source: Int J Mol Sci. 2020 Sep 22;21(18):6959. doi: 10.3390/ijms21186959 (PMC7555519; doi:10.3390/ijms21186959)
Supplement: Supplementary file 1 [file ijms-21-06959-s001.pdf]

Supplementary Materials

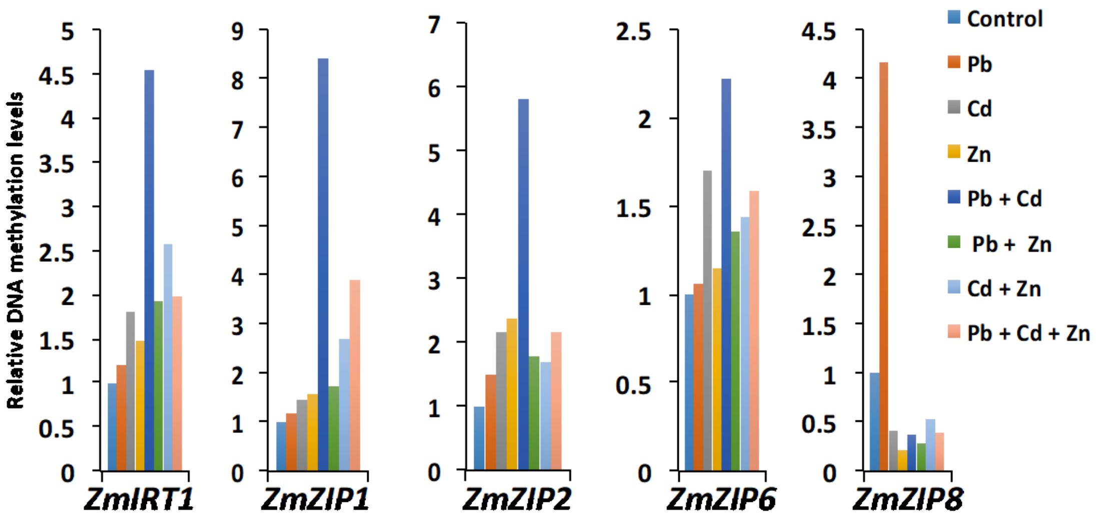

Figure 1. Quantification of DNA methylation levels at the promoter of selected *ZIP* transporters in response to Pb/Cd/Zn applied alone and in combinations from Figure 6. DNA was digested with McrBc and equal amounts of digested or undigested DNA were used as template for PCR. McrBC digests the methylated DNA; therefore, lighter band intensity reflects the more DNA methylation levels. Band intensity of digested and non-digested was calculated with Image J. To calculate the relative DNA methylation levels from the image, band intensity of non-digested was divided with McrBC digested band intensity and presented in the graph.

Table S1. Monovalent Potassium (K) in the roots, shoots and leaves in response to Pb/Cd/Zn applied alone and in combinations.

Treatment	K (mg/g)			
	Leaf	Shoot	Root	Total
Control	5.12	5.09	5.06	15.27
Pb	5.03	5.15	5.05	15.23
Cd	5.03	5.11	5.02	15.16
Zn	5.13	5.10	5.09	15.32
Pb + Cd	5.10	5.11	5.09	15.30
Pb + Zn	5.14	5.10	5.10	15.34
Cd + Zn	5.14	5.15	4.71	15.00
Pb + Cd+ Zn	5.11	5.13	5.09	15.34

The plants were grown in Hydroponic culture and metal accumulation was investigated after two weeks of treatment. The results shown are the averages of three biological replicates. Small letters represent the statistical difference ($P < 0.05$).

Table S2. Primers used in this study.

Gene		Sequence 5'-3'
qRT-PCR		
ZMZIP1	F	CCTCTCTGCGTTGGTTGCTCT
	R	TTGATGGTTGTTTTCTGGTCGT
ZMZIP2	F	CCACAAATGGCACGAGGTCT
	R	CGAAGACGGAGTGGAAGCAAA
ZMZIP3	F	GCCTCTTGTTGGTGCCCTTA
	R	TCAACAATGAACGCTGTAGTGCT
ZMZIP4	F	CCTTCTTCTCGCTCACCGCT
	R	AGCCTCGGGTTGCTGAAGT
ZMZIP5	F	GCACATAGGCATAGCCACGC
	R	ACGCCCAAAGATAGCCCGAT
ZMZIP7	F	ACTAGGTGGGTGCATTGCTCAG
	R	TGCCAGCAGATACCGAGTCAA
ZMZIP6	F	GCTACGACGATAGCAATCCAA
	R	GGTCGACTAGCGCCATATAGA
ZMZIP8	F	GTTCAAGCTGCGGTCGAT
	R	TCGTACACCGACGAGATCC
ZMIRT1	F	CATCGTGGTGGCTGACAA
	R	GACGATGCCCATCTCCAG
ZMET1	F	TTAAGCTGTCAAATGGGCAGATG
	R	CCGTACAGTCCTTTCCACTGATT
ZMMET2A	F	AAGGCTCAGCTGATAGAGAGTTG
	R	AAGAAATACCCGAAGCGGTATCA
ZMMET2B	F	TGGACAACATTGTCTACTGCCTC
	R	ACGGCAAGTAAAATAGTGGCATC
ZMMET3A	F	ACCATTGGACAAGACCAGTAGAC
	R	ACAAACTCCGGCTGAATATCGTA
ZMMET3B	F	AAGTCCCTGTAACAACCTCACTG
	R	ATGATGGAACAATGCAGAATGCTC
ZMMET3C	F	TCGTCTAGGCATCCCTTTGAAAT
	R	GTTCAGTTTTCAACCACCACCTT
ZMMET4	F	ATAGAGAAGTCCAGTCAGCCCT
	R	AAACTGTAGTCCCCAAAACCATAC
ZMHD1B	F	CGTACAATGTTCCTATGATGGTTT
	R	CAACAGCAACTGCGGTCTC
ZMHDA102	F	CTCCCAAATGAGATTCCAAAA
	R	AGGTCTTACTGTTCAAATTGTCCAT
ZMHDA110	F	AGCTGGTTCCTCAAATGGAAGAT
	R	GTAGCTGATGAGGATATCGACCG
ZMHD2A	F	AAATCTGGTGGCTCGGTCC
	R	GACTCACTTGCCCCCATCTTC
ZMHD2B	F	ATGATACCAGTGATGATGACGAGG
	R	AAAGAGGCGTTTTCAGAGCATTTT
ZMHD2C	F	CCTGAAGAAGGCGATGATGATTC
	R	GAGTTTTCAGCACGGAACTTTCA
ZMHDA106	F	TGATTCCAGTGATGAATCTGGT
	R	TTCTTGCCAACTACTACATCAGTCTT
ZMHDA1	F	CGTTGGAGCAGTTCAATGACTTT
	R	ATACACAGTGTTTCCGTCTTTGC

ZMRPD3	F	ACTGGATGATATACGATCTAAACTTTC
	R	TTCTGTGTCAGGAACTCTCTCTT
ZMUBQ	F	AACCAGCTGAGGCCCAAGA
	R	ACGATTGATTTAACCAGTCCATGA
**ChOP-PCR**		
ZMZIP1	F	GCTCTTCTTTGTTCTCGTCGTC
	R	GCAGGAGGACTCAAAGTGCCCA
ZMZIP2	F	TGGTGTTCGTCGGCACGCT
	R	TTGGCGTCGCTGAGGAAGTGC
ZMZIP6	F	TGGCTACATGATCTGAGAATGAC
	R	GATGGTTAATTTCAATGCGGTGT
ZMZIP8	F	GTGTCGTCATTTGCCAGTAATTC
	R	TTCAGCTCGTAGATTCATTAGGG
ZMIRT1	F	CTCGTCGTCGTGGTCACCATAGA
	R	GCAGGAGGACTCAAAGTGCCCA
